# *Salmonella* Type III Effector AvrA Stabilizes Cell Tight Junctions to Inhibit Inflammation in Intestinal Epithelial Cells

**DOI:** 10.1371/journal.pone.0002369

**Published:** 2008-06-04

**Authors:** Anne P. Liao, Elaine O. Petrof, Sumalatha Kuppireddi, Yun Zhao, Yinglin Xia, Erika C. Claud, Jun Sun

**Affiliations:** 1 Department of Medicine, Gastroenterology and Hepatology Division, University of Rochester, Rochester, New York, United States of America; 2 Department of Medicine, GI Diseases Research Unit and Division of Infectious Diseases, Queen's University, Kingston, Ontario, Canada; 3 Department of Paediatrics, The University of Chicago Medical Center, Chicago, Illinois, United States of America; Centre for DNA Fingerprinting and Diagnostics, India

## Abstract

*Salmonella* Typhimurium is a major cause of human gastroenteritis. The *Salmonella* type III secretory system secretes virulence proteins, called effectors. Effectors are responsible for the alteration of tight junction (TJ) structure and function in intestinal epithelial cells. AvrA is a newly described bacterial effector found in *Salmonella*. We report here that AvrA expression stabilizes cell permeability and tight junctions in intestinal epithelial cells. Cells colonized with an AvrA-deficient bacterial strain (AvrA−) displayed decreased cell permeability, disruption of TJs, and an increased inflammatory response. Western blot data showed that TJ proteins, such as ZO-1, claudin-1, decreased after AvrA- colonization for only 1 hour. In contrast, cells colonized with AvrA-sufficient bacteria maintained cell permeability with stabilized TJ structure. This difference was confirmed *in vivo*. Fluorescent tracer studies showed increased fluorescence in the blood of mice infected with AvrA- compared to those infected with the AvrA-sufficient strains. AvrA- disrupted TJ structure and function and increased inflammation *in vivo*, compared to the AvrA- sufficient strain. Additionally, AvrA overexpression increased TJ protein expression when transfected into colonic epithelial cells. An intriguing aspect of this study is that AvrA stabilized TJs, even though the other TTSS proteins, SopB, SopE, and SopE2, are known to disrupt TJs. AvrA may play a role in stabilizing TJs and balancing the opposing action of other bacterial effectors. Our findings indicate an important role for the bacterial effector AvrA in regulation of intestinal epithelial cell TJs during inflammation. The role of AvrA represents a highly refined bacterial strategy that helps the bacteria survive in the host and dampen the inflammatory response.

## Introduction

Intestinal epithelial cells participate in immune regulation and mucosal integrity. Tight junctions (TJs) constitute continuous circumferential seals around cells and serve as a protective barrier, preventing solutes and water from passing freely through the paracellular pathway. Tight junctions can be altered by various pathogens, as well as by their toxins. These effects may result from direct modification of TJ proteins such as occludin, claudin, and ZO-1, or by alteration of the perijunctional actomyosin ring [1], [2], [3].

*Salmonella enterica serovar* Typhimurium is a major cause of human gastroenteritis. Infection of polarized epithelial cell monolayers by *S.* Typhimurium disrupts TJ structure and function [4], [5], [6], [7]. TJ disruption is dependent on the type III secretory system (TTSS) of *Salmonella*. TTSS is a needle-like protein transport device used by Gram-negative pathogenic bacteria. It allows bacteria to inject virulence effectors into eukaryotic host cells [8]. TTSS is encoded by the *Salmonella* pathogenicity island 1 (SPI-1) [9]. A recent study indicated that SopB, SopE, SopE2, and SpiA are the TTSS secreted SPI-1 effectors responsible for the disruption of TJ structure and function [10]. The specific bacterial effectors responsible for the regulation of TJs, however, remains to be identified. The majority of published studies regarding *Salmonella* and TJ have utilized *in vitro* cultured epithelial models. The physiological consequences of *Salmonella*-effector-induced alteration of TJ function need to be addressed *in vivo* using animal models.

AvrA is a newly described bacterial effector transported into the host cell by the TTSS of *Salmonella* [11]. It also belongs to the SPI-1 [11]. The SPI-1 effectors are responsible for early inflammation in the mouse model of *S*. Typhimurium-induced enterocolitis [12], [13]. AvrA protein from *Salmonella* Typhimurium inhibits activation of the proinflammatory NF-κB transcription factor in cultured human epithelial cells [14]. Based on the sequence alignment, AvrA belongs to the cysteine protease family [15]. Representative AvrA members include the adenovirus-like proteases (human adenovirus type 2, fowl adenovirus 8, *Hemorrhagic enteritis* virus), YopJ (Yersinia outer protein J), and AvrBsT. The catalytic triad for the cysteine protease is present in all AvrA family members [15], [16]. Further studies demonstrated that expression of a mutant AvrA protein with a single amino acid residue transition (AvrA/C186A) in a putative catalytic cysteine of this enzyme did not inhibit TNFα-stimulated induction of the reporter [14]. We recently demonstrate that AvrA has deubiquitinase activity which removes ubiquitins from ub-IκBα, thus inhibiting NF-κB activity [17]. AvrA C186A mutant protein had reduced deubiquitinase activity as evidenced by cleaving less ubiquitin moieties from IκBα [17]. This data further supports the hypothesis that AvrA protein has protease activity which attenuates the proinflammatory NF-κB pathway.

The *AvrA* gene is present in 80% of *Salmonella enterica serovars* [18]. The protein expression of AvrA differs strikingly between bacterial strains in systemic disease and in enteritis which is localized to the intestine [18]. AvrA protein was not expressed in strains related to systemic disease, but was conditionally (pH below 6.0) expressed in the enteritis-related strains. In addition, *S. enterica* strains from systemic infections could be characterized by their strong SopB and SopE1 expression and by the absence of SopD1 and AvrA proteins [18]. Ben-Barak *et al.* [19] identified four phenotypic classes of *S. enterica* under defined standard culture conditions: strains with a constitutive synthesis of AvrA; strains with an acid induction of AvrA; strains with silent *avrA* genes; and a fourth class without *AvrA* gene [19]. Taken together, AvrA protein expression is very different from the other *Salmonella* effectors such as SopB, SopD, and SopE [19]. Although it is premature to claim a correlation of AvrA with the clinical and epidemiological potency of *Salmonellae*, current studies indicate that a fine-tuning of AvrA expression takes place during the pathogenesis of *Salmonella* infection.

Unlike SopB and SopD, AvrA does not increase physiologic fluid secretion into infected calf ileal loops [20],[21]. However, the role of AvrA expression on the tight junction structure and function of the intestinal epithelial cells in both *in vitro* and *in vivo* models is unexplored. To determine if AvrA was responsible for the TJ protein expression and distribution, we focused on bacterial strains sufficient or deficient in AvrA—parental PhoP^c^, PhoP^c^ AvrA mutant (AvrA−) or the AvrA complementary strain (PhoP^c^ AvrA−/AvrA+). PhoP^c^ is a PhoP-PhoQ constitutive mutation of a WT *Salmonella* Typhimurium strain 14028s that increases the expression of PhoP-activated genes, represses the synthesis of approximately 20 proteins encoded by the PhoP-repressed genes, and attenuates virulence [22]. Reed *et al.*, showed that PhoP^c^ has similar adherence ability as the WT *Salmonella* and is less invasive than the WT *Salmonella* using the MDCK and T84 cell models [23]. A previous study demonstrated that PhoP^c^ is able to inhibit the activation of the proinflammatory NF-κB pathway [24]. Further study showed that AvrA expression in PhoP^c^ plays an importance role in attenuating the NF-κB activity by stabilizing IκBα, the inhibitor of NF-κB [14] [17]. In current study, we continue to use this *Salmonella*-epithelial interaction system.

We hypothesized that AvrA expression in *Salmonella* Typhimurium is able to regulate the TJ protein expression and distribution in intestinal epithelial cells, and hence, change TJ structure. We have tested *Salmonella* Typhimurium with AvrA sufficient or deficient expression in a cultured polarized human epithelial cell model, an animal model, and in AvrA-transfected cells. We demonstrate that *Salmonella* lacking AvrA decreased tight junction protein expression in both cultured colonic epithelial cell and bacterial infected mouse models. While examining changes in resistance and cell permeability, we also investigated TJ protein expression, as well as effects on TJ protein distribution induced by AvrA-deficient and -sufficient bacterial strains *in vitro* and *in vivo*. Our data demonstrate that TJ protein expression increased significantly in cells transiently transfected with the *AvrA* gene. Our findings suggest an important role for the bacterial effector AvrA in regulating the structure and function of tight junctions in intestinal epithelial cells.

## Materials and Methods

### Cell culture

T84 epithelial cells (American Type Culture Collection, Manassas, VA) were grown in 1:1 DMEM and Ham's F-12 medium supplemented with 15 mM HEPES (pH 7.5), 14 mM NaHCO3, antibiotics, and 5% neonatal calf serum. HT29-CL19A cells were grown in DMEM (high glucose, 4.5 g/L) containing 5% (vol/vol) fetal bovine serum, 50 ug/ml streptomycin, and 50 U/ml penicillin. Monolayers of T84 and HT29-CL19A cells were grown on permeable supports (0.33 or 4.67 cm^2^, 0.4 µm pore. Costar, Cambridge, MA) and utilized 6–14 days (T84) or 4–6 days (HT-29-CL19A) after being plated.

### Bacterial strains and growth conditions

Bacteria strains included wild-type (WT) *S*. Typhimurium ATCC 14028s; *S.* Typhimurium PhoP^c^, a derivative of wild-type *Salmonella* SL14028 [22] with AvrA gene and protein expression; *Salmonella* PhoP^c^ mutant strain lacking the AvrA gene (PhoP^c^ AvrA−); PhoP^c^ AvrA- transcomplemented with a plasmid encoding WT AvrA (PhoP^c^ AvrA−/AvrA+) [14]; and *Escherichia coli* F18 (a flagellated nonpathogenic strain [25], [26]). *S. typhimurium* mutant PhoP^c^, PhoP^c^ AvrA-, and PhoP^c^ AvrA−/AvrA+ were provided by Dr. Andrew Neish of Emory University. The wild-type strain *Salmonella* ATCC 14028s used in our study is known to have the *AvrA* gene but has low AvrA protein expression [18], [19]. Wild-type *S. typhimurium* AvrA+ was generated by transforming with the pWSK29-AvrA plasmid and ampcillin-resistance selected. Bacterial growth conditions were as follows: non-agitated microaerophilic bacterial cultures were prepared by inoculation of 10 ml of Luria-Bertani broth with 0.01 ml of a stationary phase culture, followed by overnight incubation (∼18 h) at 37°C, as previously described [25]. Bacterial overnight cultures were concentrated 33-fold in Hank's balanced salt solution (HBSS) supplemented with 10 mM HEPES, pH 7.4.

### Bacterial colonization in the polarized epithelial cells *in vitro*

Polarized human colonic epithelial cells were colonized with equal numbers of the indicated bacteria for 30 min, washed with HBSS, and incubated in DMEM containing gentamicin (500 µg/ml) for the times indicated in our previous study [17], [27]. The first 30-minute incubation allowed bacteria to contact the surface of the epithelial cells and inject the effectors in the host cells. After extensive HBSS washing, the extracellular bacteria were washed away. Incubation with gentamicin inhibited the growth of bacteria. In this way, we focused on the effects of the bacterial effectors injected to the host cells.

### Streptomycin pre-treated mouse model

Animal experiments were performed using specific-pathogen-free female C57BL/6 mice (Taconic) that were 6–7 weeks old. The protocol was approved by the University of Rochester Committee on Animal Recources Water and food were withdrawn 4 h before oral gavage with 7.5 mg/mouse of streptomycin (75 µl of sterile solution or 75 µl of sterile water [control]). Afterwards, animals were supplied with water and food *ad libitum*. Twenty hours after streptomycin treatment, water and food were withdrawn again for 4 hours before the mice were infected with 1×10^7^ CFU of *S. typhimurium* (50-µl suspension in HBSS) or treated with sterile HBSS (control) by oral gavage as previously described [25]. At 6, 18, and 24 hours after infection, mice were sacrificed and tissue samples from the intestinal tracts were removed for analysis.

### Immunoblotting

Mouse epithelial cells were scraped and lysed in lysis buffer (1% Triton X-100, 150 mM NaCl, 10 mM Tris pH 7.4, 1 mM EDTA, 1 mM EGTA pH 8.0, 0.2 mM sodium ortho-vanadate, protease inhibitor cocktail) and protein concentration measured. T84 or HT29-CL19A Cells were colonized with equal numbers of the indicated bacteria for 30 minutes, washed with HBSS, and incubated in DMEM containing gentamicin (500 µg/ml) for the times indicated. Cells were lysed in protein loading buffer (50 mM Tris, pH 6.8, 100 mM dithiothreitol, 2% SDS, 0.1% bromphenol blue, 10% glycerol). Equal volumes of total cell lysate were separated by SDS-PAGE, transferred to nitrocellulose, and processed for immunoblotting with Mouse anti-α-catenin, Rabbit anti-claudin-1, Mouse anti-occludin-1, Mouse anti-ZO-1 antibodies from Zymed Laboratories Inc.(South San Francisco, CA), or E-cadherin antibodies from BD Transduction Laboratories( Franklin Lakes, NJ).

### Immunoblotting for AvrA

Bacteria were lysed in lysis buffer [in mM: 50 Tris, pH 8.0, 150 NaCl, 5 EDTA with a complete Mini protease inhibitor cocktail (1 tablet/10 ml, Roche), and 1% Triton X-100], and sonicated. Equal amounts of total proteins were loaded, separated by SDS-PAGE, and processed for immunoblotting with custom-made AvrA antibody. The 15 amino acid (aa) peptide CGEEPFLPSDKADRY was designed based on the AvrA sequence aa#216-230 (GenBank accession no. AE008830).

### AvrA transfection

HT29CL19A cells were grown in 12-well plates. At 70–80% confluence, cells were transfected with a pCMV-*myc-AvrA* wild-type gene construct, a pCMV-*myc-AvrAC186A* AvrA mutant construct, or control empty pCMV-*myc* plasmid using LipofectAMINE (Invitrogen). The AvrA mutant C186A is a single amino acid residue transition which is mutated at the key cysteine required for AvrA's activity as previously described [14], [17]. 24 h after transfection, cells were lysed in protein loading buffer (50 mM Tris, pH 6.8, 100 mM dithiothreitol, 2% SDS, 0.1% bromophenol blue, 10% glycerol). Equal volumes of total cell lysate were separated by SDS-PAGE, transferred to nitrocellulose, and processed for immunoblotting.

### Immunofluorescence staining

Cultured epithelial cells T84 or HT29-CL19A were incubated with equal numbers of the indicated bacteria for 30 minutes and washed with HBSS. Immunofluorescent labeling of cells grown on inserts was performed as follows: cells were fixed for 30 minutes in 1% paraformaldehyde in PBS and then washed in PBS. Fixed samples were incubated in blocking solution (5% bovine serum albumin, 0.1% saponin, 1 mM calcium in PBS) for 20 minutes, followed by a 90 minute incubation with primary antibodies diluted in blocking solution (1% bovine serum albumin, 0.1% saponin, 1 mM calcium in PBS): 1:100 Rabbit anti-Claudin-1 (Zymed Laboratories Inc., South San Francisco, CA); 1:1000 Mouse anti-ZO-1 (Zymed Laboratories Inc., San Francisco, CA). After a 60 minute incubation with secondary antibodies: 1:200 Alexa Fluor 488 goat-anti-rabbit IgG H+L; 1:200 Alexa Fluor 594 goat-anti-mouse IgG H+L; 1:10,000 4′,6-diamidino-2-phenyl-indole, dihydrochoride (DAPI) (all from Molecular Probes, Eugene, OR), the inserts were mounted with SlowFade (SlowFade® AntiFade Kit, Molecular Probes) followed by a coverslip, and the edges were sealed to prevent drying. Specimens were examined with a Leica SP2 A OBS Laser Scanning confocal microscope.

Colonic tissues from the proximal and distal portion of the colon were freshly isolated and embedded in paraffin wax after fixation with 10% neutral buffered formalin. After preparation of the slides as described above, slides were incubated in 3% H_2_O_2_ for 20 minutes at room temperature to block endogenous peroxidase activity, followed by incubation for 20 min in 5% BSA with 0.1% saponin in PBS to reduce nonspecific background. The samples were incubated with primary antibodies as indicated for 90 minutes at room temperature. Samples were then incubated with goat anti-rabbit Alexa Fluor 488 (Molecular Probes, Invitrogen Detection Technologies, Eugene, OR, USA; 1:200), goat anti-mouse Alexa Fluor 594 (Molecular Probes, CA, USA; 1:200), and DAPI (Molecular Probes 1:10 000) for 1 h at room temperature. Tissues were mounted with SlowFade. Specimens were examined with a Leica SP2 A OBS Laser Scanning confocal microscope.

### TER measurement

Cells were grown as monolayers on collagen-coated polycarbonate membrane Transwell supports (Corning-Costar, Acton, MA). Cells were colonized with equal numbers of the indicated bacteria for 30 minutes, washed with HBSS, and incubated in DMEM containing gentamicin (500 µg/ml, Invitrogen Corporation) for the time indicated. Transepithelial resistance (TER) was measured with an epithelial voltohmmeter (EVOM, World Precision Instruments, Sarasota, FL). Each measurement was performed in triplicate.

### Fluorescence Permeability *in vivo*

Streptomycin pre-treated mice were infected with different bacterial strains for 24 hours. Fluorescein Dextran (Molecular weight 3000 Da, diluted in HBSS) was gavaged (50 mg/g mouse). Four hours later, mouse blood samples were collected by cardiac puncture. Fluorescence intensity of the plasma was measured on a fluorescent plate reader [28].

### Statistical Analysis

Data are expressed as mean±SD. Differences between two samples were analyzed by Student's t test. P-values of 0.05 or less were considered significant.

## Results

### AvrA expression alters tight junction protein expression in human epithelial cells

**Figure 1:**
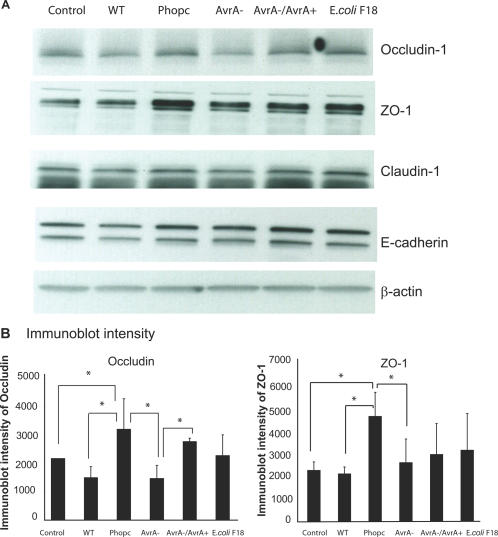
AvrA expression stabilizes the protein expressions of occludin and ZO-1 *in vitro*. (A) Western blot of occludin-1, ZO-1, claudin-1, and E-cadherin. Polarized human colonic epithelial T84 cells were colonized with AvrA-deficient or -sufficient bacterial strains for 30 minutes, washed with HBSS and incubated in DMEM for 30 minutes. Cells were lysed. Equal volumes of total cell lysate were processed for immunoblotting with Rabbit anti-claudin-1, Mouse anti-occludin-1, Mouse anti-ZO-1 antibodies, or E-cadherin antibodies. Experimental groups: Control: polarized T84 cells without any treatment; WT: wild-type *S*. Typhimurium ATCC 14028s without sufficient AvrA protein expression; PhoP^c^: parental PhoP^c^ with sufficient AvrA protein expression; AvrA^−^: PhoP^c^ AvrA mutant; AvrA^−^/AvrA^+^: PhoP^c^ AvrA- transcomplemented with a plasmid encoding WT AvrA; or *E.coli* F18: commensal bacteria isolated from human intestine. (B) Densitometry of occludin and ZO-1. Relative occludin-1 and ZO-1 band intensity was determined using NIH Image 1.63 software. Occludin-1 and ZO-1 expression significantly increased in the PhoP^c^ group compared to the Control, WT, and AvrA- groups in absence of AvrA protein. * P<0.05. Data are reported as mean±SD of 3 independent experiments.

First, we analyzed whether infection of T84 cell monolayers with AvrA protein-sufficient or -deficient bacterial strains could influence the expression of the major proteins which comprise the tight junction complex. We assessed the expression of the tight junction proteins, claudin-1, occludin-1, and Zonula occludens-1 (ZO-1) by Western blot. We also tested the adhesion protein E-cadherin. After bacterial colonization in epithelial cells for only one hour, both the wild-type *S. Typhimurium* 14028s (with insufficient AvrA expression) and the PhoP^c^ AvrA mutant strain lacking the AvrA gene (PhoP^c^ AvrA−) led to a down-regulation of the TJ proteins ZO-1, occludin, and claudin-1 ( Fig. 1A). In contrast, the parental PhoP^c^ with sufficient AvrA expression stabilized TJ protein expression. *E.coli* F18 failed to modulate the expression of occludin-1 and claudin-1, which is consistent with the report by Köhler *et al*. [26]. In Fig. 1B, the immunoblot intensity analysis demonstrated that occludin and ZO-1 expression was significantly increased by the presence of PhoP^c^ with AvrA protein expression, whereas the AvrA-deficient strain (AvrA−) and wild-type *Salmonella* 14028s with insufficient AvrA protein induced a significantly less in ZO-1 and occludin expression. AvrA expression also stabilized TJ proteins in HT-29CL19A monolayers (data not shown).

### AvrA overexpression in epithelial cells increases TJ protein expression

**Figure 2:**
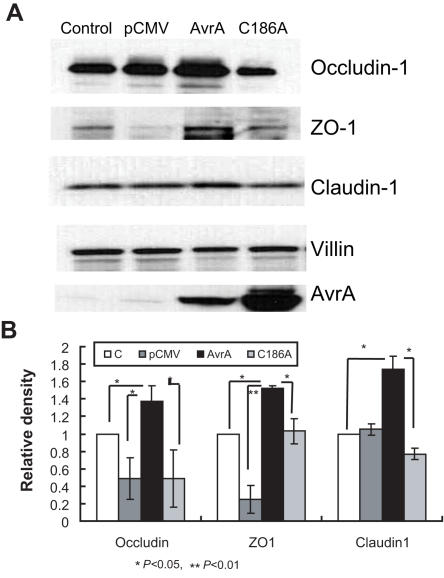
AvrA transfection in epithelial cells increases TJ protein expression. (A) HT29C19A cells were transfected with a pCMV-*myc-AvrA* wild-type gene construct, a pCMV-*myc-AvrAC186A* AvrA mutant construct, or control empty pCMV-*myc* plasmid using LipofectAMINE (Invitrogen). The AvrA mutant C186A is a single amino acid residue transition which is mutated at the key cysteine required for AvrA activity. 24 h after transfection, cells were lysed in protein-loading buffer. Equal volumes of total cell lysis were processed for immunoblotting for ZO-1, occludin-1, claudin-1, AvrA, and β-actin. Control: normal HT29Cl19A without treatment; pCMV: cells transfected with empty pCMV-*myc* plasmid; AvrA: cells transfected with pCMV-*myc-AvrA* plasmid; C186A: cells transfected with AvrA mutant C186A plasmid. (B) Densitometry of ZO-1, occludin-1, and claudin-1. These are significant increase of ZO-1 and occludin-1 expression in AvrA-overexpressed cells compared to the cells without AvrA expression. AvrA mutant C186A expression did not increase ZO-1 and occludin-1 expression. It indicates that cysteine mutation abolished the effects of wild-type AvrA on TJ protein expression. * P<0.05, ** P<0.01. Data are reported as mean±SD of 2–3 independent experiments.

To determine whether AvrA expression directly regulates TJ protein, we transfected human colonic epithelial HT29CL19A cells with a pCMV-*myc-AvrA* wild-type gene construct, a pCMV-*myc-AvrAC186A* AvrA mutant construct, or a control pCMV-*myc* plasmid. The AvrA mutant C186A is a single amino acid residue transition which is mutated at the key cysteine required for AvrA activity as previously described [14], [17]. As shown in Fig. 2, AvrA overexpression in colonic epithelial cells increased ZO-1, claudin-1, and occludin-1 expression significantly, whereas the AvrA mutant C186A was able to reverse the effect and decrease the TJ protein expression to the levels comparable to those in the cells transfected with empty pCMV-*myc* vector. These data indicate that AvrA expression directly increases TJ protein expression. The cysteine site required for the AvrA activity is involved in AvrA regulation of TJ protein expression.

### AvrA expression alters tight junction protein distribution *in vitro*

We further examined tight junction protein distribution. Epithelial cells colonized with AvrA-sufficient or -deficient strains were analyzed for the location of claudin-1 and ZO-1.

**Figure 3:**
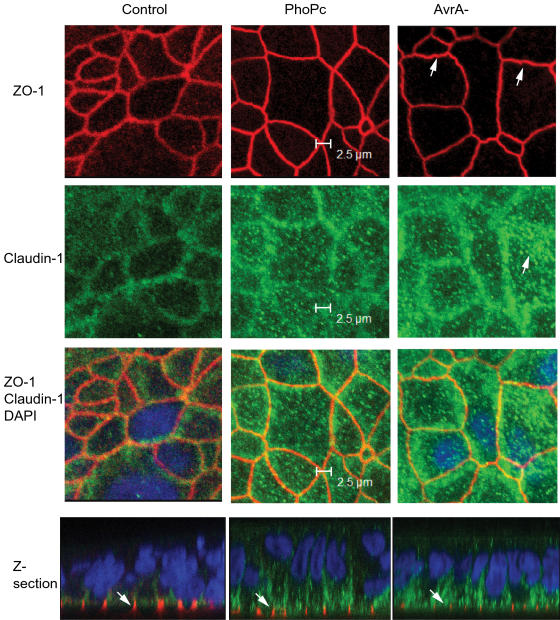
Immunostaining of claudin-1 and ZO-1 in cells colonized with AvrA-sufficient or -deficient bacteria *in vitro.* T84 monolayers were treated with PhoPc or AvrA-. After 8 hours, the monolayers were fixed and immunostained for claudin-1 and ZO-1. ZO-1 distribution in the control cells without any treatment has its normally smooth nature. In PhoP^c^-treated cells, the distribution of ZO-1 was very similar to that in the control cells. ZO-1's appearance in PhoP^c^ group was similar as the control group when cells were viewed in cross-Z-section (Z-section for Control and PhoP^c^). However, in cells treated with *Salmonella* derivative AvrA- mutant (without AvrA), the normally smooth arc-like ZO-1 profiles were transformed into a complex series of irregular undulations (first row of panels AvrA-). ZO-1 staining became thinner and more sinuous. The Z-section panel in Fig. 3 shows the weak staining of ZO-1 in AvrA-. AvrA absence induced a disorganization of transmembrane protein claudin-1, and the protein was moreover expanded intracellularly (second row, see arrow). PhoP^C^ treatment also slightly changed the distribution of claudin-1. Intracellular claudin-1 was detectable in the cytosol of the cells colonized with PhoP^c^. Results are representative of 5 independent experiments.

ZO-1: ZO-1 is a cytoplasmic plaque tight junction protein. In control monolayers without any treatment, ZO-1 was restricted to cellular borders and distributed in a smooth arc-like pattern. In PhoP^c^ treated cells, the distribution of ZO-1 was very similar to that in the control cells. The appearance of ZO-1 in the PhoP^c^ group was similar to the control group when cells were viewed in cross-Z-section (Figure 3. Z-section for Control and PhoP^c^). However, in cells treated with *Salmonella* derivative AvrA- mutant (without AvrA), the normally smooth arc-like ZO-1 profiles were transformed into a complex series of irregular undulations (Fig. 3 first row of panels AvrA−). Further, ZO-1 staining became thinner and more sinuous. The Z-section panel in Fig. 3 shows the weak staining of ZO-1 in AvrA−.

Claudin-1: Claudin-1 is highly enriched at sites of cell-cell contact, co-localizing with the TJ marker, ZO-1 [29]. AvrA absence induced a disorganization of transmembrane protein claudin-1, and the protein was expanded intracellularly (Fig. 3 second row, see arrow). Interestingly, PhoP^C^ treatment also slightly changed the distribution of claudin-1. Intracellular claudin-1 was detectable in the cytosol of the cells colonized with PhoP^c^. This indicated that additional bacterial proteins may be involved in regulating TJs. Overall, our immunofluorescence data suggest that AvrA modulates junctional localization of ZO-1 and claudin-1 proteins.

### TER and AvrA expression

Transepithelial resistance (TER) is a measure of intestinal epithelial integrity and tissue viability [30], [31]. We assessed the TER of the epithelial cells before and after bacterial colonization. Cells were colonized with AvrA-sufficient or -deficient bacterial strains for 30 minutes and then washed. TER of monolayers was measured after switching to fresh media containing gentamicin to prevent further bacterial growth. Our data showed that the baseline TER (Ω cm^2^) at 0 minute in controls without treatment was 987.1±6.8 Ω cm^2^. The TER values for cultured epithelial cells from the control group remained relatively stable over the 30 to 90 minute incubation period. There was a decrease of TER (482.1±5.3 Ω cm^2^) after AvrA- colonization for 30 minutes, whereas parental PhoP^c^, a derivative of wild-type *Salmonella* SL14028s, did not change TER significantly. It is consistent with previous study that SL14028s did not have effect on the TER of T84 cells [25]. In our study, the TER change was focus in the initial 6 hours. Overall, cells colonized with the AvrA-deficient bacterial strain (AvrA−) had the lowest TER compared to the control, PhoP^c^, and PhoPc AvrA+/ AvrA− groups, but there was no significant difference among the groups(data not shown).

### AvrA expression and permeability *in vivo*

**Figure 4:**
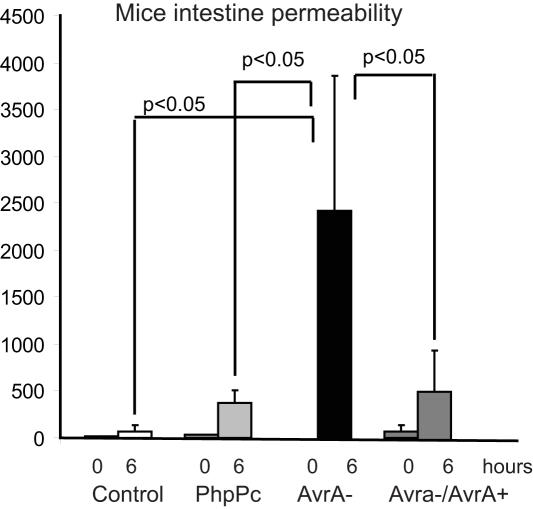
AvrA regulated permeability in the human colonic epithelial cells. Permeability of the intestine *in vivo*. Data are representative of three experiments. *P*<.05 for control vs. AvrA-, PhoP^C^ vs. AvrA-, and AvrA- vs. AvrA−/+ after infection for 28 hours.

To assess the biological relevance of AvrA expression *in vivo*, we utilized a streptomycin-pretreatment mouse model [13] and gavaged the mice with parental PhoP^c^, AvrA-, or PhoP^c^ AvrA−/AvrA+ strains. Immunofluorescence-tagged FITC-dextran was also gavaged in each mouse for the permeability assay (Fig. 4). Mouse serum was collected to measure the intensity of fluorescence. Higher FITC readings indicate higher permeability of the intestine. There was a 5-fold increase of the fluorescence reading in the AvrA- infected mouse serum compared to that in the PhoP^c^ mouse serum. In the PhoP^c^ AvrA−/AvrA+ group, complemented AvrA expression was able to significantly decrease cell permeability. Our data demonstrated that AvrA-sufficient bacteria significantly decrease the intestinal permeability compared to AvrA-deficient bacteria. It indicates the physiological function of AvrA in preserving intestinal epithelial cell integrity *in vivo*.

### AvrA expression stabilizes the expression of the tight junction proteins *in vivo*

**Figure 5:**
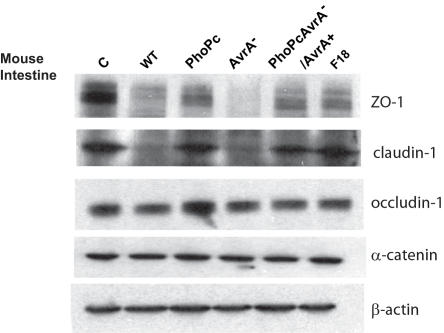
The *Salmonella* AvrA protein modulated ZO-1, occludin, and claudin-1 expression *in vivo*. Mice were infected with *bacteria* for 18 hrs and intestinal epithelial cells were harvested for ZO-1, claudin-1, occludin, and α-catenin expression by immunoblot. Experimental groups: C: normal mouse cells; WT: wild-type *S*. Typhimurium ATCC 14028s without sufficient AvrA protein expression; PhoP^c^: parental PhoP^c^ with sufficient AvrA protein expression; AvrA^−^: PhoP^c^ AvrA mutant; PhoP^c^ AvrA^−^/AvrA^+^: PhoP^c^ AvrA- transcomplemented with a plasmid encoding WT AvrA; or *E.coli* F18: commensal bacteria isolated from human intestine. Images shown are from a single experiment and are representative of three separate experiments.

We collected the epithelial cells from mouse colon and quantitated the TJ protein expression (Fig. 5). As expected, wild-type *Salmonella* 14028s colonization decreased the total amount ZO-1, claudin-1, and occludin-1 protein expression. AvrA- decreased ZO-1, claudin-1, and occludin-1 expression, whereas total occludin-1 expression was increased by the parental PhoP^c^ strain with AvrA expression. Interestingly, the claudin-1 expression was stabilized but not increased by the PhoP^c^ colonization. In the PhoP^c^ AvrA−/AvrA+ group with complemented AvrA, the expression of ZO-1, claudin-1, and occludin-1 was stabilized to levels comparable to those in the samples from the parental PhoP^c^-treated group. Compared to the normal mice without bacterial infection, all the bacterial infected colonic epithelial cells had decrease ZO-1 expression. In a cell cultured model, *E.coli* F18 infection failed to change the expression of TJ proteins (Fig. 1). However, *E.coli* F18 infection *in vivo* decreased ZO-1 expression. This suggests that other bacterial proteins are involved in the regulation of ZO-1 expression *in vivo*. Interestingly, wild-type *Salmonella* and *E.coli* F18 infection did not change the expression of α-catenin *in vivo*. AvrA has no effect on the expression of α-catenin.

### AvrA expression changes the distribution of tight junction proteins *in vivo*

Immunostaining of ZO-1 and claudin-1 in the experimental animal models further showed that parental PhoP^c^ with AvrA expression maintained TJ structure in the epithelial cells.

**Figure 6:**
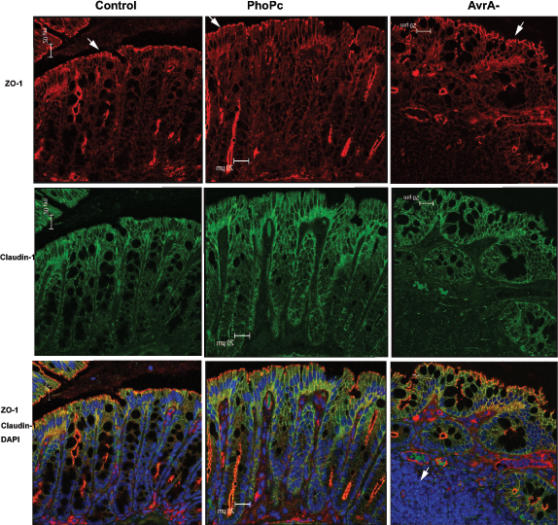
Immunostaining of claudin-1 and ZO-1 *in vivo*. Immunostaining on mouse colonic epithelial cells was performed 24 hours after mouse infection with PhoP^c^, AvrA^−^ or AvrA^−^/AvrA^+^. Experimental groups: Control: normal mouse cells; PhoP^c^: mice infected with parental PhoP^c^ with sufficient AvrA protein expression; AvrA^−^: mice infected with PhoP^c^ AvrA mutant. Tissues were fixed, permeabilized, and stained with claudin-1 and ZO-1 antibodies, followed by A488 secondary antibodies, A594 secondary antibodies, and DAPI. AvrA^−^ infected mice display disruption of the TJ structure. Arrows in Panel ZO-1 show the red staining of ZO-1 protein on the top of the intestinal crypts. White arrow in Panel AvrA- DAPI shows lymphoid aggregation. Please note disorganized structure of ZO-1 in the colonic epithelial cells infected with AvrA- bacterial strain. Images shown are from a single experiment and are representative of three separate experiments. *n* = 3 animals in each experimental group.

**Figure 7:**
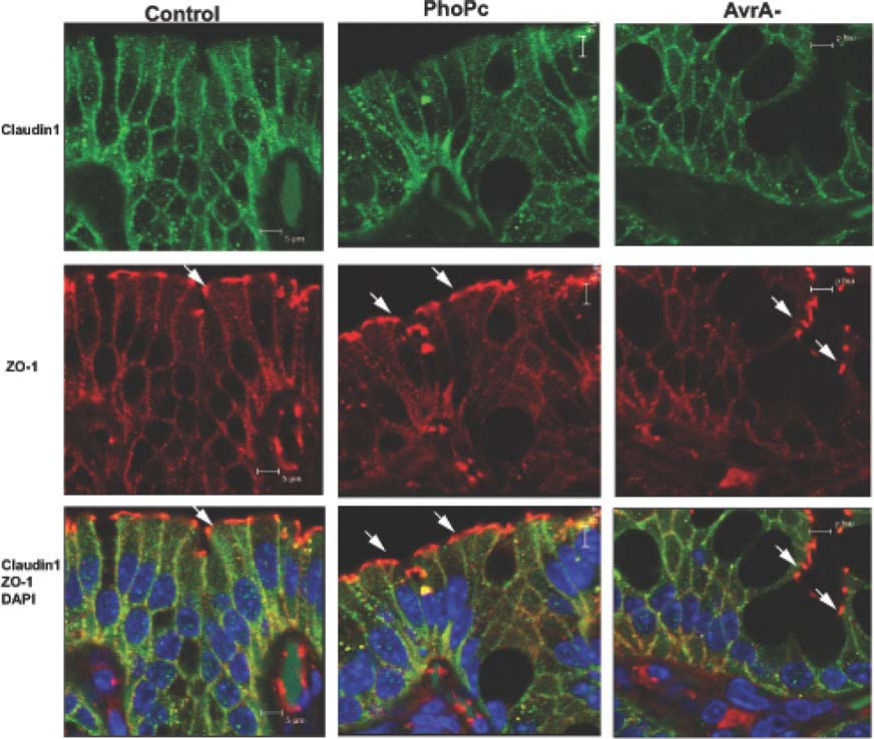
Higher magnification of claudin-1 and ZO-1 immunostaining *in vivo*. Arrows in Panel ZO-1 show red staining of ZO-1 protein on the top of the intestinal crypts. Panel AvrA^−^ showing disrupted ZO-1 and weaker claudin-1 staining in epithelial cells. ZO1 was detected at the tight junction of villous enterocytes in both normal control and PhoP^c^–treated animals. No intracellular ZO-1 deposits were detected after PhoP^c^ infection. The ring-like structure of ZO-1 was disrupted in mouse colon infected by the AvrA-deficient bacteria. The staining of claudin-1 is weaker in the AvrA- treated intestinal epithelium. No intracellular claudin-1 deposits were detected after PhoP^c^ or AvrA- infection. *n* = 3 animals in each experimental group.

ZO-1: ZO1 was detected at the tight junction of villous enterocytes in both normal control and PhoP^c^–treated animals. Intracellular ZO-1 deposits were not detected after PhoP^c^ infection. Under low magnitude observation in Fig. 6, we found that the AvrA-deficient mutant disrupted the TJ structure, whereas parental PhoP^c^ with AvrA protein expression stabilized the TJ structure. Arrows in Fig. 6 ZO-1 show the red staining of ZO-1 protein on the top of the intestinal crypts. Please note the disorganized structure of ZO-1 in the colonic epithelial cells infected with the AvrA- bacterial strain. Under high magnification observation in Fig. 7: the ring like structure of ZO-1 was disrupted in mouse colon infected by the AvrA-deficient bacteria.

Claudin-1: The staining of green claudin-1 is weaker in the AvrA- treated intestinal epithelium. Intracellular claudin-1 deposits were not detected after PhoP^c^ or AvrA- infection. These *in vivo* data combined with *in vitro* data (Fig. 3) indicate that additional bacterial proteins may be involved in regulating the distribution of the TJ proteins. Overall, our immunofluorescent data suggested that AvrA modulates junctional localization of ZO-1 and claudin-1 proteins.

Also, in Fig. 6AvrA- with ZO-1, Claudin-1 overlapped DAPI staining; there was increased inflammation in the epithelial cells as measured by lymphoid aggregation, whereas the tight junction structure was disrupted. Our H & E staining indicated that AvrA absence in the bacterial strain (AvrA-) increased the inflammation score in the infected intestine (data not shown). In the mice infected with parental PhoP^c^, the tight junction structure was still well organized, and there was less inflammation in the intestine.

### AvrA protein expression attenuates IL-6 secretion

**Figure 8:**
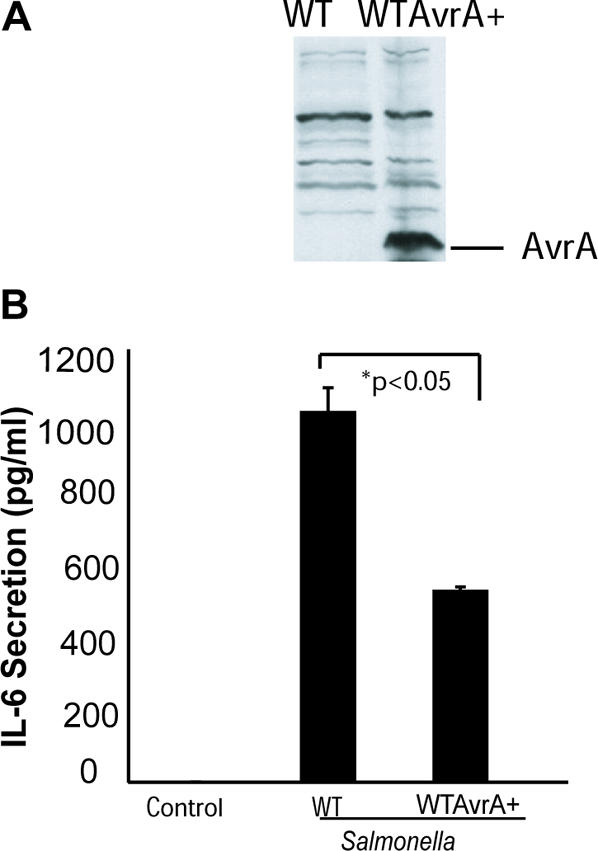
*Salmonella* effector AvrA inhibited IL-6 secretion in mice. (A) AvrA protein expression level in the AvrA-sufficient or -deficient bacterial strains. Total bacterial lysates were immunoblotted with antibodies against AvrA. (B) IL-6 levels in mouse serum samples 2 hours after WT *Salmonella* or WT *Salmonella* AvrA infection. Data shown in (B) are *mean*±*SD* for n = 3 animals in each experimental group. Significance was at p≤0.05.

It is known that cells colonized with AvrA-sufficient bacteria lack inflammatory response [17]. AvrA may stabilize TJ structure by dampening the inflammatory response. To assess the biological relevance of AvrA expression *in vivo*, we infected mice with WT *Salmonella* Typhimurium strain 14028s (WT) with insufficient AvrA expression or WT 14028s with AvrA overexpression (WTAvrA+). As shown in Fig. 8A, AvrA protein expression is undetectable in WT *Salmonella*14028s, whereas WTAvrA+ showed a significant increase in AvrA expression. We measured the inflammatory cytokine IL-6 in mouse serum after bacterial infection. WT *Salmonella* induced significantly more IL-6 secretion as measured in infected mouse serum than did the WTAvrA+ (Fig. 8B). In mice infected with the WTAvrA+, AvrA overexpression was able to lower IL-6 serum levels. It is suggested that AvrA expression in the WT *Salmonella* is able to decrease the expression of inflammatory cytokine IL-6.

## Discussion

Our data demonstrate that the bacterial effector protein AvrA stabilizes the expression and distribution of tight junction proteins such as ZO-1, and the function of tight junctions *in vitro* and *in vivo*. AvrA overexpression in transfected colonic epithelial cells increases TJ protein expression. Bacterial strains with AvrA stabilize host cell permeability, cell adhesion, and tight junction and inhibit the inflammatory response. In contrast, AvrA-deficient strains induce morphological and biochemical changes, including increased cell permeability, disrupted TJ structure, and inflammatory responses.

An intriguing aspect of this study is the finding that AvrA stabilized the TJs, whereas the other TTSS proteins, SopB, SopE, SopE2, and SpiA, are known to disrupt the TJs [10]. Although initially this observation appears unusual, it may represent a highly refined bacterial strategy to overcome many effective host defense mechanisms. Previous studies have demonstrated that AvrA does not stimulate fluid secretion into infected calf ileal loops, whereas SopB and SopD elevate fluid accumulation in bovine intestine [20]. Current studies show that lack of AvrA increases the cell permeability and disrupted TJ structure, whereas AvrA expression is able to maintain the TJ structure and function and limit the cell permeability. Our data on AvrA stabilization of TJ structure and permeability suggest a different role for AvrA distinct from the role of other *Salmonella* effectors in regulating fluid accumulation in intestine.

*Salmonella* effectors, such as SopB, SopE, SopE2, are know to activate the proinflammatory response by directly stimulating proinflammatory signaling events in host cells [32], [33], [34], [35]. In contrast, AvrA is able to attenuate the key proinflammatory NF-κB transcription factor [14], [17], activate the β-catenin transcription factor [27], [36], and inhibit cell apoptosis in mouse epithelial cells [17]. Therefore, AvrA may function as an anti-inflammatory protein to stabilize TJs, prevent cell death, and help the bacteria survive in the host; whereas the other bacterial effectors do the opposite. Further investigation is needed to determine whether AvrA influences TJ by damping the inflammatory response in the host epithelial cells. Characterization of the synergistic functions of AvrA with other bacterial effectors and toxins will give new clues concerning microbial–host interaction in inflammation.

The PhoP^c^ we used is a derivative of wild-type *Salmonella* Typhimurium SL14028. Previous studies indicated that infection with wild type SL14028 did not influence TER [25], whereas recent studies using the SL1344 showed different results [10], [26]. Several factors explain these differences. First, the *S.* Typhimurium background of these strains is different. Since the SL1344 strain induces a more robust response in the ability to induce PMN transepithelial migration than the 14028 strain, and this differences in the virulence phenotype could explain, in part, differences at the level of the TER [26]. Second, the level of AvrA expression by a particular *Salmonella* strain may ultimately determine how that organism will behave. Wild type *Salmonella* strains express AvrA conditionally, but at levels insufficient to counteract the actions of other bacterial agents. *S*L14028 does not have detectable AvrA protein [18], [19], whereas the SL1344 sufficiently expresses AvrA protein [11]. Therefore, the TER was not changed by infection with SL14028, whereas it was changed by infection with SL1344.

We have found that expression of occludin-1, claudin-1, and ZO-1 are altered by AvrA expression using a gene-transfected system, cultured polarized epithelial cells, and a mouse model. Based on our data, AvrA likely has a specific role in the expression of ZO-1 and occludin. The key 186 amino acid cysteine is required for AvrA regulation of TJ expression. However, it is not clear whether AvrA regulates these TJ proteins through phosphorylation or through ubiquitination. AvrA acts as a deubiquitinase to inhibit the degradation of the inflammatory regulators IκBα and β-catenin [17]. Occludin is a functional target of the E3 ligase Itch [37]. Thus, AvrA may stabilize TJ protein by removing ubiquitin from occludin. Rho GTPase is known to be involved in bacteria-induced tight junction disruption [10], [38], [39], [40], [41]. Our current study demonstrates that AvrA is able to stabilize the TJ structure, but it is unclear whether Rho GTPase is influenced by the AvrA expression. Future studies will explore these questions and provide further insight into bacteria-host interactions during inflammation. Taken together, our data and the findings of others indicate that bacteria, in order to survive in the host, have evolved multiple approaches of hijacking host epithelial cells.
